# Addiction and Choice: Theory and New Data

**DOI:** 10.3389/fpsyt.2013.00031

**Published:** 2013-05-06

**Authors:** Gene M. Heyman

**Affiliations:** ^1^Department of Psychology, Boston CollegeBoston, MA, USA

**Keywords:** addiction, choice theory, remission, correlates of recovery, brain disease model

## Abstract

Addiction’s biological basis has been the focus of much research. The findings have persuaded experts and the public that drug use in addicts is compulsive. But the word “compulsive” identifies patterns of behavior, and all behavior has a biological basis, including voluntary actions. Thus, the question is not whether addiction has a biology, which it must, but whether it is sensible to say that addicts use drugs compulsively. The relevant research shows most of those who meet the American Psychiatric Association’s criteria for addiction quit using illegal drugs by about age 30, that they usually quit without professional help, and that the correlates of quitting include legal concerns, economic pressures, and the desire for respect, particularly from family members. That is, the correlates of quitting are the correlates of choice not compulsion. However, addiction is, by definition, a disorder, and thereby not beneficial in the long run. This is precisely the pattern of choices predicted by quantitative choice principles, such as the matching law, melioration, and hyperbolic discounting. Although the brain disease model of addiction is perceived by many as received knowledge it is not supported by research or logic. In contrast, well established, quantitative choice principles predict both the possibility and the details of addiction.

## Introduction

Addictive drugs change the brain, genetic studies show that alcoholism has a substantial heritability, and addiction is a persistent, destructive pattern of drug use (e.g., Cloninger, 1987; American Psychiatric Association, 1994; Robinson et al., 2001). In scientific journals and popular media outlets, these observations are cited as proof that “addiction is a chronic, relapsing brain disease, involving compulsive drug use” (e.g., Miller and Chappel, 1991; Leshner, 1999; Lubman et al., 2004; Quenqua, 2011). Yet, research shows that addiction has the highest remission rate of any psychiatric disorder, that most addicts quit drugs without professional help, and that the correlates of quitting are those that attend most decisions, such as financial and familial concerns (e.g., Biernacki, 1986; Robins, 1993; Stinson et al., 2005; Klingemann et al., 2010). However, addiction is “disease-like” in the sense that it persists even though on balance its costs outweigh the benefits (e.g., most addicts eventually quit). Thus, in order to explain addiction, we need an account of voluntary behavior that predicts the persistence of activities that from a global bookkeeping perspective (e.g., long-term) are irrational. That is, addiction is not compulsive drug use, but it also is not rational drug use. Several empirical choice principles predict the possibility of relatively stable yet suboptimal behavior. They include the matching law, melioration, and hyperbolic discounting (e.g., Herrnstein, 1990; Ainslie, 1992). These principles were discovered in the course of experiments conducted in laboratories and natural settings, and in experiments these same principles also distinguish addicted from non-addicted drug users (e.g., Kirby et al., 1999). For example, ex and current heavy drug users were more likely to suboptimally “meliorate” than were non-addicts in a choice procedure that invited both long-term maximizing and melioration (Heyman and Dunn, 2002). Thus, we have on hand a research based, non-disease account of the defining features of addiction, which is to say its destructive and irrational aspects. As this essay is based on how those we call addicts behave, it would be most efficient to begin with a brief summary of key aspects of the natural history of addiction.

## Likelihood of Remission and Time Course of Addiction

**Figure 1:**
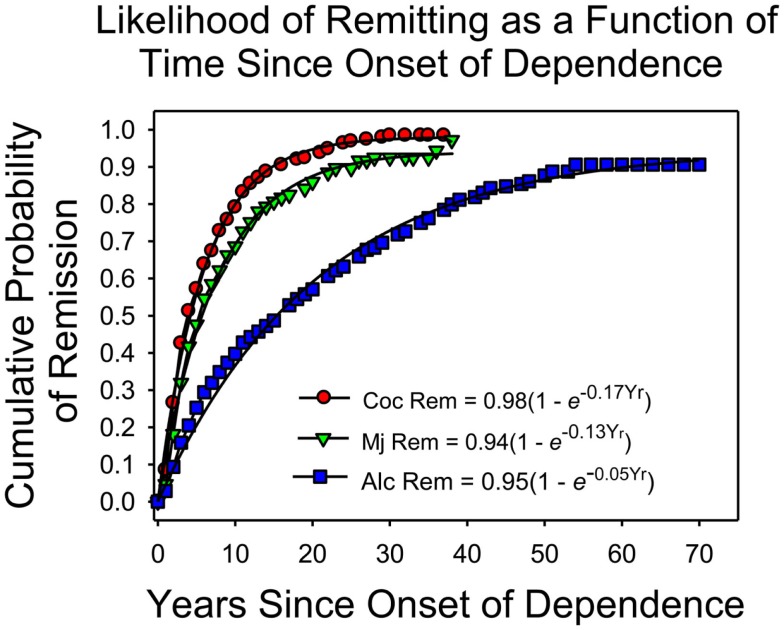
**The cumulative frequency of remission as a function of time since the onset of dependence, based on Lopez-Quintero et al.’s (2011) report**. The proportion of addicts who quit each year was approximately constant. The smooth curves are based on the negative exponential equations listed in the figure.

Figure 1 shows the cumulative frequency of remission as a function of the onset of dependence in a nation-wide representative sample of addicts (United States, Lopez-Quintero et al., 2011). The researchers first recruited a sample of more than 42,000 individuals whose demographic characteristics approximated those of the US population for individuals between the ages of 18 and 64 (Grant and Dawson, 2006). The participants were interviewed according to a questionnaire designed to produce an APA diagnosis when warranted. For those who currently or in the past met the criteria for “substance dependence” (the APA’s term for addiction), there were additional questions aimed at documenting the time course of clinically significant levels of drug use. Figure 1 summarizes the findings regarding remission and the duration of dependence.

On the *x-*axis is the amount of time since the onset of dependence. On the *y*-axis is the cumulative frequency of remission, which is the proportion of individuals who met the criteria for lifetime dependence but for the past year or more had been in remission. The fitted curves are negative exponentials, based on the assumption that each year the likelihood of remitting remained constant, independent of the onset of dependence (Heyman, 2013).

The cumulative frequency of remission increased each year for each drug. Indeed, the theoretical lines so closely approximated the observations that the simplest account is that each year a constant proportion of those who had not yet remitted did so regardless of how long they had been addicted. By year 4 (since the onset of dependence) half of those who were ever addicted to cocaine had stopped using cocaine at clinically significant levels; for marijuana the half-life of dependence was 6 years; and for alcohol, the half-life of dependence was considerably longer, 16 years. As the typical onset age for dependence on an illicit drug is about 20 (Kessler et al., 2005a), the results say that most people who become addicted to an illicit drug are “ex-addicts” by age 30. Of course, addicts may switch drugs rather than quit drugs, but other considerations indicate that this does not explain the trends displayed in Figure 1. For example, dependence on any illicit drug decreases markedly as a function of age, which would not be possible if addicts were switching from one drug to another (Heyman, 2013).

The graph also shows that there is much individual variation. Among cocaine users, about 5% continued to meet the criteria for addiction well into their 40s; among marijuana users, about 8% remained heavy users well into their 50s, and for alcoholics, more than 15% remained heavy drinkers well into their 60s. Thus, for both legal and illegal drugs some addicts conform to the expectations of the “chronic disease” label. However, as noted below, the correlates of quitting drugs are the correlates of decision making, not the correlates of the diseases addiction is said to be similar to.

## Can We Trust the Data?

The results in Figure 1 replicate the findings of previous nation-wide surveys and targeted studies that selected participants so as to obtain representative samples (e.g., Robins and Murphy, 1967; Anthony and Helzer, 1991; Robins, 1993; Warner et al., 1995; Kessler et al., 2005a,b). For instance, in every national scientific survey of mental health in the United States, most of those who met the criteria for dependence on an illicit drug no longer did so by age 30, and addiction had the highest remission rate of any other psychiatric disorder. However, research on remission faces well-known methodological pitfalls. Those in remission may relapse at some post-interview date, and the subject rosters of the large epidemiological studies may be biased in favor of those addicts who do quit. For instance, addicts who remain heavy drug users may not cooperate with researchers or may be hard to contact because of their life style, illnesses, or have higher mortality rates. These issues have been discussed in some detail elsewhere (Heyman, 2013). The key results were that remission after age 30 was reasonably stable, and that it was unlikely that there were enough missing or dead addicts to alter significantly the trends displayed in Figure 1.

## The Correlates of Quitting and the Role of Treatment

The correlates of quitting include the absence of additional psychiatric and medical problems, marital status (singles stay addicted longer), economic pressures, fear of judicial sanctions, concern about respect from children and other family members, worries about the many problems that attend regular involvement in illegal activities, more years spent in school, and higher income (e.g., Waldorf, 1983; Biernacki, 1986; Waldorf et al., 1991; Warner et al., 1995). Put in more personal terms, addicts often say that they quit drugs because they wanted to be a better parent, make their own parents proud of them, and not further embarrass their families (e.g., Premack, 1970; Jorquez, 1983). In short, the correlates of quitting are the practical and moral concerns that affect all major decisions. They are not the correlates of recovery from the diseases addiction is said to be like, such as Alzheimer’s, schizophrenia, diabetes, heart disease, cancer, and so on (e.g., Leshner, 1999; McLellan et al., 2000; Volkow and Li, 2004).

Much of what we know about quitting drugs has been provided by researchers who study addicts who are not in treatment (e.g., Klingemann et al., 2010). This is because most addicts do not seek treatment. For instance, in the survey that provided the data for Figure 1, only 16% of those who currently met the criteria for dependence were in treatment, and treatment was broadly defined so as to include self-help organizations as well as services by trained clinicians (Stinson et al., 2005). Since most addicts quit, the implication is that most addicts quit without professional help. Research supports this logic (e.g., Fiore et al., 1993).

## A Non-Disease Etiology for Persistent Self-Destructive Drug Use

Although self-destructive, irrational behavior can be a sign of pathology, it need not be. The self-help industry is booming, which reflects the tendency of so many of us to procrastinate, overeat, skip exercising, and opt for whatever is most convenient. Why buy a book or go to a lecture on how to improve your life if you did not realize that (1) you were behaving imprudently, (2) knew you probably could change, but (3) so far have not taken the requisite steps. Similarly, human irrationality drives the story-line of most novels, memoirs, movies, and plays. Agamemnon sacrifices his own daughter to advance his political and personal goals but then publicly embarrasses Achilles his most powerful and skillful warrior. Both actions are selfish, and the second undermines the goals of the first, which anyone could have foretold. However, Homer is portraying human nature not writing a psychiatric text. Thus, it seems fair to say that who cite selfishness and myopic choices as evidence of pathology (e.g., “she has to be sick because she bought drugs rather than groceries”) naively misread human nature.

In support of the poet’s as opposed to the brain disease account of human nature, behavioral psychologists and economists have discovered principles that predict self-defeating, selfish patterns of behavior. They include “hyperbolic discounting,” “melioration,” and the “matching law” (Herrnstein, 1970, 1990; Rachlin and Green, 1972; Ainslie, 1992; Rachlin, 2007). These are quantitative, empirical laws of choice that predict how different species, including humans, choose between different commodities and activities, such as food, water, and exercise. Their relevance to addiction and other self-defeating behaviors is that under some conditions they predict relatively stable yet suboptimal patterns of behavior. For example, Heyman and Herrnstein (1986) arranged an experiment in which the matching predicted the lowest possible rate of reinforcement. As predicted the subjects shifted to matching, lowering their overall reinforcement rate as they did so. This finding has been replicated numerous times (e.g., Herrnstein et al., 1997), and it is analogous as to what happens as drug use turns into addiction.

Or, put another way, general principles that apply to everyday choices, also predict compulsive-like consumption patterns that are consistent with the behavior of addicts.

These choice laws reflect a basic, but often overlooked property, of most choice situations. There is more than one “optimal” strategy (Heyman, 2009). One is optimal from the perspective of the most immediate circumstances, such as the current values of the options, taking into account just the most pressing needs and goals. The others are optimal in terms of wider time horizons and the perspectives of others. For example, in settings in which current choices affect the values of future options, it is possible for the current best choice to be the worst long-term choice (e.g., Herrnstein et al., 1993; Heyman and Dunn, 2002). This is relevant because a common feature of addictive drugs is that they provide immediate benefits but delayed costs. Thus, it is possible that the drug is the best choice when the frame of reference is restricted to the current values of the immediately available options but the worst choice when the frame of reference expands to include future costs and other people’s needs. According to this account, persistent drug use reflects the workings of a local optimum, whereas controlled drug use or abstinence reflects the workings of a global optimum. Put somewhat differently, whether or not drug use persists depends on the factors that influence decision making, particularly values that emphasize global as opposed to a local frame of reference (e.g., values related to family, the future, one’s reputation, and so on). Scores of studies support this analysis (e.g., Waldorf, 1983; Biernacki, 1986; Mariezcurrena, 1994; Klingemann et al., 2010).

## Old Clinical Follow-Up Studies: Empirical Support for the Disease Account

Imagine that what we knew about addiction was restricted to those individuals who make up the right-hand tails of the cumulative distribution curves in Figure 1. We would have good reason to believe that addiction is a chronic relapsing disease. This is precisely the situation for much of the history of addiction research. Until the mid 1970s virtually all empirical studies of addicts were based on individuals who had been in treatment, which was most often detoxification in American prison/hospitals or similar institutions (e.g., Brecher, 1972; Vaillant, 1973; Maddux and Desmond, 1980; Hser et al., 1993). In some studies virtually all of the participants were males with extensive arrest records, poor work histories, lower than average marriage rates, and lower than average educational achievement (e.g., Vaillant, 1973). That is, the understanding of addiction as a chronic disorder was based on a population of drug users whose demographic characteristics – we now know – match those that predict not quitting (e.g., Klingemann et al., 2010). In the 1960s illicit drug use spread to college campuses and upscale neighborhoods. This new generation of addicts included individuals who were employed, married, and well-educated (e.g., Waldorf et al., 1991). With these demographic changes, the natural history of addiction changed. More often than not, the pressures of family, employment, and the hassles of an illegal life style eventually trumped getting high. Figure 1, which is representative of every major epidemiological study conducted over the past 30 years, reflects this reality; received opinion does not.

## But Drugs Change the Brain

With the exception of alcohol, addictive drugs produce their biological and psychological changes by binding to specific receptor sites throughout the body. As self-administered drug doses greatly exceed the circulating levels of their natural analogs, persistent heavy drug use leads to structural and functional changes in the nervous system. It is widely – if not universally – assumed that these neural adaptations play a causal role in addiction. In support of this interpretation brain imaging studies often reveal differences between the brains of addicts and comparison groups (e.g., Volkow et al., 1997; Martin-Soelch et al., 2001) However, these studies are cross-sectional and the results are correlations. There are no published studies that establish a causal link between drug-induced neural adaptations and compulsive drug use or even a correlation between drug-induced neural changes and an increase in preference for an addictive drug. For example, in a frequently referred to animal study, Robinson et al. (2001) found dendritic changes in the striatum and the prefrontal cortex of rats who had self-administered cocaine. They concluded that this was a “recipe for addiction.” However, they did not evaluate whether their findings with rodents applied to humans, nor did they even test if the dendritic modifications had anything to do with changes in preference for cocaine in their rats. In principle then it is possible that the drug-induced neural changes play little or no role in the persistence of drug use. This is a testable hypothesis.

First, most addicts quit. Thus, drug-induced neural plasticity does not prevent quitting. Second, in follow-up studies, which tested Robinson et al.’s claims, there were no increases in preference for cocaine. For instance in a preference test that provided both cocaine and saccharin, rats preferred saccharin (Lenoir et al., 2007) even after they had consumed about three to four times more cocaine than the rats in the Robinson et al study, and even though the cocaine had induced motoric changes which have been interpreted as signs of the neural underpinnings of addiction (e.g., Robinson and Berridge, 2003). Third, Figure 1 shows that the likelihood of remission was constant over time since the onset of dependence. Although this is a surprising result, it is not without precedent. In a longitudinal study of heroin addicts, Vaillant (1973) reports that the likelihood of going off drugs neither increased nor decreased over time (1973), and in a study with rats, Serge Ahmed and his colleagues (Cantin et al., 2010) report that the probability of switching from cocaine to saccharin (which was about 0.85) was independent of past cocaine consumption. Since drugs change the brain, these results suggest that the changes do not prevent quitting, and the slope of Figure 1 implies that drug-induced neural changes do not even decrease the likelihood of quitting drugs once dependence is in place.

## But There is a Genetic Predisposition for Addiction

Twin and adoption studies have repeatedly demonstrated a genetic predisposition for alcoholism (e.g., Cloninger, 1987), and the limited amount of research on the genetics of illicit drug use suggests the same for drugs such as heroin, cocaine, and marijuana (Tsuang et al., 2001). However, all behavior has a genetic basis, including voluntary acts. The brain is the organ of voluntary action, and brain structure and development follow the blueprint set by DNA. Thus, there is no necessary connection between heritability and compulsion. In support of this point, monozygotic twins are much more likely to share similar religious and political beliefs than are dizygotic twins, even when they are separated before the age of 1 year old (e.g., Waller et al., 1990; McCourt et al., 1999). That is, learned, voluntary religious and political beliefs have substantial heritabilities just as do many involuntary human characteristics. The relevance to addiction is that a genetic predisposition is not a recipe for compulsion, just as brain adaptations are not a recipe for compulsion.

## Summing Up

Addiction involves an initial “honey moon” period, followed by alternating periods of remission and relapse, and then an eventual return to a more sober life. Most addicts quit using drugs at clinically significant levels, they typically quit without professional help, and in the case of illicit drugs, they typically quit before the age of 30. The correlates of quitting include many of the factors that influence voluntary acts, but not, according to Figure 1, drug exposure once drug use meets the criteria for dependence. Thus, we can say that addiction is ambivalent drug use, which eventually involves more costs than benefits (otherwise why quit?). Behavioral choice principles predict ambivalent preferences, semi-stable suboptimal behavior patterns, and the capacity to shift from one option to another. In contrast, the brain disease account of addiction fails to predict the high quit rates; it fails to predict the correlates of quitting; it fails to predict the temporal pattern of quitting; and it is tied to unsupportable assumptions, such as the claims that neural adaptations, heritability, and irrationality are *prima facie* evidence of disease. To be sure “compulsion” and “choice” can be seen as points on a continuum, but Figure 1 and research on quitting make it clear that addiction is not a borderline case.

It is time to think about addiction in terms of what the research shows, particularly the more recent epidemiological studies, and it is time to abandon the medical model of addiction. It does not fit the facts. The matching law, melioration, and hyperbolic discounting predict that drugs and similar commodities will become the focus of destructive, suboptimal patterns of behavior. These same choice models also predict that individuals caught in a destructive pattern of behavior retain the capacity to improve their lot and that they will do so as a function of changes in their options and/or how they frame their choices. This viewpoint fits the facts of addiction and provides a practical guide to measures that will actually help addicts change for the better.

## Conflict of Interest Statement

The authors declare that the research was conducted in the absence of any commercial or financial relationships that could be construed as a potential conflict of interest.
